# Individual Variation Exists Within the Psychological Response to Hypoxic Bed Rest: A Retrospective Analysis

**DOI:** 10.3389/fphys.2022.810055

**Published:** 2022-02-10

**Authors:** Kunihito Tobita, Igor B. Mekjavic, Adam C. McDonnell

**Affiliations:** ^1^Department of Sustainable System Sciences, Osaka Prefecture University, Sakai, Japan; ^2^Department of Automation, Biocybernetics and Robotics, Jožef Stefan Institute, Ljubljana, Slovenia; ^3^Department of Biomedical Physiology and Kinesiology, Simon Fraser University, Burnaby, BC, Canada

**Keywords:** individual variability, hypoxia, bed rest, psychology, emotion, psychological trajectory

## Abstract

Individual variation is of interest to Space Agency’s, which cannot be explored with astronauts due to anonymity. We retrospectively analysed data collected throughout three projects (LunHab: 10-day male, PlanHab: 21-day male, and FemHab: 10-day female) to elucidate the potentially masked individual variation in the psychological responses to bed rest. The Profile of Mood State (POMS) and Positive and Negative Affect Schedule (PANAS) – instruments used to asses psychological state – and Lake Louise Mountain Sickness (LLMS) scores were collected prior to, following and throughout three interventions: 1: normoxic bed rest 2: hypoxic bed rest and 3: hypoxic ambulatory confinement. Total Mood Disturbance (TMD) was calculated from the POMS results, positive affect (PA), and negative affect (NA) from PANAS. The three instruments were included in a latent class mixed model. TMD, NA, and LLMS were included in a four-class model, with each class representing a specific type of response (Class 1: descending, Class 2: flat, Class 3: somewhat flat, Class 4: ascending). Responses for PA were assigned to only two classes (Classes 1 and 2). 54.55% or 24 participants were included in Class 2 (TMD, NA, and LLMS), where the responses did not change and neither hypoxia or activity level had a significant effect on emotional state. The remaining participants were allotted to Class 1, 3, or 4, where hypoxia was a significant covariate, while activity (bed rest) was significant only for class 3. For PA, 84.09% or 37 participants were assigned to class 2 indicating a significant effect of hypoxia on the participants responses with no effect of physical activity. Class 1 participants (*n* = 7) were not affected by hypoxia, however, physical activity improved their PA. Participants undergoing confinement, hypoxia and bed rest do not exhibit a uniform emotional response and may be categorised into 2–4 distinct classes. These results indicate significant individual emotional responses, that may be masked and underreported by traditional statistical approaches like means ± SD. The emotional state of our participants is a complex construct likely influenced by past experiences and different coping mechanisms which allowed some to adapt to the experimental environment more readily.

## Introduction

Extra vehicular activity (EVA) on the International Space Station (ISS) has taken place on average once every month over the last 20 plus years of flight. While ISS is a phenomenal aerospace and research platform, it has become clear that future long-term space exploration will establish permanent habitats on the Moon and Mars rather than continue a singular reliance on low earth orbit research. One critical difference between the current (ISS) and future (planetary) platforms will be the rate of EVAs, which are expected to be daily or more often. Currently, the pressure inside space suits is about one third of the normobaric (1 atmosphere) pressure inside ISS. Decompression sickness mitigation protocols are utilised in the preparation for EVAs and are not compatible with daily use. If one were to move freely between, for example, a Martian habitat and the external environment (in an EVA or extrahabitat activity suit; EHA) with the current ambient, the risk of decompression sickness would be too great. In order to minimise the astronauts’ risk of such, future habitats will likely be hypobaric and hypoxic (Norcross et al., 2005, 2013; Bodkin et al., 2006) allowing for safe and straightforward ingress and egress. While the effect of bed rest – as a reduced gravity analogue – in normoxia has been extensively studied, the addition of hypoxia in combination with bed rest has been a core research idea of our laboratory for more than a decade.

A reduction in oxygen supply to the central nervous system may result in symptoms of altitude sickness, such as headache, nausea, fatigue, and weakness (Matthys, 2011) along with a variety of neuropsychological impairments (Bahrke and Shukitt-Hale, 1993; Davidson, 2001) that includes alterations in cognition, mood, behaviour, and sleep indices (Winget and DeRoshia, 1986; Hornbein, 2001). Additionally, acute hypoxic exposure reduces exercise performance by 18% (Deb et al., 2018) and worsens sleep efficiency and memory performance (de Aquino Lemos et al., 2012). The degree of performance loss varies greatly depending on the measurement item. With regard to personality, undesirable changes may occur, namely increased paranoia and obsessive compulsiveness (Nelson, 1982). Given that emotional stability enhances physical adaptation to altitude in terms of fatigue and acute mountain sickness symptoms (Virues-Ortega et al., 2004), it would be important to clarify the effects of hypoxic exposure on one’s psychological state. Further, it may provide useful insights into the smooth operation of communities in enclosed spaces. Additionally, it should be noted that decreased gravitational load and physical activity levels have been shown to affect cognitive and psychomotor functioning (Kanas et al., 2009; Lipnicki and Gunga, 2009; Basner et al., 2021). Some of those studies have reported that the participants’ emotions changed negatively (Ishizaki et al., 2002; Liu et al., 2012), while others have reported no change (Zhao et al., 2011). With regard to spaceflight, the conclusion of one review (Strangman et al., 2014) points to small sample sizes and effect sizes that provide weak support for cognitive changes and as such, there is a need to better understand the influence of individual variability on cognitive performance.

More recently, the European Space Agency (ESA) has indicated an interest in the individual variation exhibited in physiological and psychological responses to bed rest, microgravity and space flight. There are several sources of error or variability within an experimental study, particularly in a descriptive or observational experiment compared to a mechanistic intervention. The primary point of variation to be considered is the precision and accuracy of the measurement tool, secondly, inherent random effects of the participants and finally, the observer effect, that which you examine or measure you alter. While the mean or median is useful in describing the expected outcome of the intervention, it does not accurately describe the response of all individuals within the experimental group or take into account these sources of error. Therefore, it becomes important to quantify the given individual response in order to further understand the expected outcome. Individual differences in emotional reactivity and emotional style have been identified as manifesting in the peak amplitude of the response, the rise time to the peak, and the recovery time (Davidson, 1998, 2001). Unfortunately, because hypoxia and inactivity studies require sophisticated research facilities and great effort on the part of both participants and experimenters, the sample size is typically relatively small and individual differences cannot be tested. As such, we sought to combine the results from three near identical research projects to establish the degree of variability in the psychological state of the individuals after the interventions. For the readers interest and specific comparison between the styles of data presentation, one is referred to previous publications from our group (Stavrou et al., 2015, 2018a,2018b) where some of the data used in the current retrospective analysis has been published as means and standard deviations.

This was the impetus for the current work, which set out to reanalyse the results of the profile of mood state (POMS), Positive and Negative Affect Schedule (PANAS), instruments of emotional state and Lake Louise Mountain Sickness (LLMS) which were collected during the Slovene Bed Rest Programme.

## Materials and Methods

The European Space Agency (ESA) established a programme of research to collect and reanalysis bed rest data conducted in the Slovene Bed Rest Programme. The current manuscript retrospectively analysed data collected during that programme. The purpose in doing so was to evaluate the extent of individual variability evident in the psychological response to bed rest, confinement and/or hypoxia. The projects included were: LunHab – 10-day male Lunar Habitat Simulation; PlanHab – 21-day male Planetary Habitat Simulation; and FemHab – 10-day female Planetary Habitat Simulation. All experimental procedures were conducted according to the ESA bed rest standardisation recommendations (Standardisation of bed rest study conditions 1.5, August 2009) and conformed to the Declaration of Helsinki. The study protocols were approved by the National Committee for Medical Ethics at the Ministry of Health of the Republic of Slovenia; approval numbers: 205/2/11 and 88/04/12.

### Participants

Inclusion and exclusion criteria were applied according to the standard operating procedures set out by ESA (Heer et al., 2009). Fifteen males, fourteen males and fifteen females took part in the LunHab, PlanHab, and FemHab projects, respectively. Participants were recreationally active lowland Slovene residents (<500 m). The baseline characteristics of the participants are outlined in Table 1.

**TABLE 1:** Baseline characteristics of the participants.

	LunHab	PlanHab	FemHab
	
*N*	15	14	15
Age, year	24.1 ± 2.2	26.4 ± 5.0	26.1 ± 3.6
Stature, cm	179.2 ± 7.6	179.5 ± 5.0	168.4 ± 6.0*
Body mass, kg	71.9 ± 10.9	76.9 ± 10.4	59.6 ± 8.2*
BMI, kg/m^2^	22.4 ± 2.8	23.8 ± 2.7	21.0 ± 2.3
VO_2_ max, mL/kg/min	43.3 ± 5.5	44.3 ± 6.1	41.0 ± 3.8

*BMI, Body mass index; V̇O_2_ max, maximal volume of oxygen uptake.*

*Significance between projects.

### Study Outline

The detailed study protocols have been described elsewhere (McDonnell et al., 2019, 2020). Briefly, 9 study campaigns were conducted in the hypoxic facility at the Olympic Sports Centre Planica (Rateče, Slovenia) situated at an altitude of 940 m under the Slovene Bed Rest Programme. Each experimental campaign comprised baseline and recovery periods – before and after the intervention – so that the participants commitment to the project was 33 days in PlanHab and 18 days in both LunHab and FemHab. Each participant underwent three interventions in a cross-over randomised design manner: normobaric normoxic [fraction of inspired O_2_, F_*I*_O_2_: 0.209; partial pressure of inspired oxygen (P_*I*_O_2_): 133 mmHg] horizontal bed rest (NBR); normobaric hypoxic (F_*I*_O_2_: 0.142; P_*I*_O_2_: 91 mmHg, target simulated altitude of 4,000 m) horizontal bed rest (HBR); and normobaric hypoxic (F_*I*_O_2_: 0.142; P_*I*_O_2_: 91 mmHg) ambulatory confinement (HAMB). The interventions were separated by at least 4 weeks for the washout period (4 weeks in LunHab; 2 months in FemHab; 4 months in PlanHab) to allow for the effects of the prior exposure to hypoxia and/or inactivity to be eliminated. With the exception of length of interventions and participant sex, the protocols of the three interventions were similar in all three projects, thus allowing for the comparison of the data. Complete bed rest schedules have been published for LunHab (McDonnell et al., 2019) and FemHab (McDonnell et al., 2020).

During the bed rest interventions (NBR and HBR), no deviations from the lying position, muscle stretching or static contractions were permitted. Participants in the HAMB condition were allowed to move freely within the hypoxic area and engaged in two 30-min bouts of daily aerobic physical activity. The purpose of this physical activity was to mimic the level and amount of daily activity that the participants would normally perform outside of the present project and not to induce a training stimulus. Adherence to the assigned protocol was ensured using continuous closed-circuit television surveillance and constant supervision by the research and medical staff.

### Measurements

#### Profile of Mood States

Participants completed the Profile of Mood States – Short Form (POMS; Shacham, 1983) at regular time points throughout the interventions. POMS consists of a list of 37 adjectives with a 5-point Likert scale. Participants reported their subjective mood states on the questionnaire ranging from 0 “not at all” to 4 “extremely.” Total Mood Disturbance (TMD) was calculated based on the results given to the questionnaire by the participants.

#### Positive and Negative Affect Schedule

The Positive and Negative Affect Schedule is a self-report mood scale that can measure positive affect (PA) and negative affect (NA) separately (Watson et al., 1988). The Instrument consists of two 10-item mood words, and participants rated the extent to which they experienced each of the emotions described in PANAS. Participants answered each item based on a 5-point scale with anchors of “very slightly or not at all” (1) to “very much” (5), with intermediate points of 2 representing “a little,” 3 “moderately,” and 4 “quite a bit.” The total score for each affect factor (PA and NA) ranging from 10 to 50 was calculated by summating the 10-items’ score.

#### Lake Louise Mountain Sickness

The Lake Louise Mountain Sickness self-report questionnaire (Roach et al., 1993) was completed daily to assess for the presence of acute mountain sickness (AMS). The respondents’ rate five symptoms (headache, gastrointestinal upset, fatigue/weakness, dizziness/light-headedness, and sleep disturbance) on a scale of 0–3 for severity, with a total score of 3 or higher and the presence of headache considered a diagnosis of AMS. The rating for each symptom is accompanied by a brief description of the severity of the symptom.

The POMS, PANAS, and LLMS data collection schedules are provided in Table 2 for each of the three projects.

**TABLE 2:** Measurement day overview.

	POMS			PANAS			LLMS		
	FemHab	LunHab	PlanHab	FemHab	LunHab	PlanHab	FemHab	LunHab	PlanHab
PRE	x	x	x	x	x	x	x		x
D1	x		x	x		x	x	x	x
D2							x	x	x
D3							x	x	x
D4							x	x	x
D5	x	x		x	x		x	x	x
D6							x	x	x
D7			x			x	x	x	x
D8							x	x	x
D9							x	x	x
D10	x	x		x	x		x	x	x
D11									x
D12									x
D13									x
D14			x			x			x
D15									x
D16									x
D17									x
D18									x
D19									x
D20									x
D21			x			x			x
POST	x	x	x	x	x	x	x		x

*POMS, Profile of Mood States; PANAS, Positive and Negative Affect Schedule; LLMS, Lake Louise mountain sickness.*

### Statistical Analysis

#### Background

Latent classes mixed modelling (lcmm) for multivariate longitudinal markers (Proust et al., 2006; Proust-Lima et al., 2013) was used to identify trajectories of the participants psychological state during the interventions. Instead of treating all individuals as those that share the same psychological profiles across time, lcmm may identify unmeasured latent classes that represent subgroups of participants with similar psychological trajectories. A series of psychological data sets were assessed using the multlcmm function of the lcmm package version 1.7.9 (Proust-Lima et al., 2017) in R, version 3.5.0 (R Core Team, 2020). The model estimation is based on a robust maximum likelihood and can handle longitudinal data series with intermittent data missing at random (Proust and Jacqmin-Gadda, 2005; Proust-Lima et al., 2017). No participant had missing values for any of the covariates (please see below). The linear, quadratic, and cubic models were tested for each day that data was collected in all interventions (HBR, NBR, HAMB) in order to calculate the coefficients of TMD, Negative affect and LLMS. The lcmm that included PA as a dependent variable did not converge, as a result, PA was analysed with a univariate model and thus presented separately. As aforementioned, the data collection days were not identical across the three bed rest projects; to allow the quadratic and cubic terms in the model to adequately process the values and align the time variable labels, the day of the intervention when data was collected was transformed from PRE, D1, D2…, POST to whole numbers and then divided by the campaign duration and finally multiplied by 100 to give a percentage value.

#### Covariates

Sex, F_*I*_O_2_ (normoxia or hypoxia), intervention duration (10 or 21 days) and activity level (bed rest or ambulatory) were entered as covariates within the model. Sex and intervention duration were not significant, and as such the model had a poor “goodness of fit” and therefore these variables were excluded as covariates. The significance of each of these factors was assessed with the multivariate Wald test (Wald, 1943). Random effects for intervention day, F_*I*_O_2_ and activity level all reduced the models’ goodness of fit, thus the intercept was included in the model as a random effect on an individual level.

#### Procedures

Following the recommended approaches for lcmm (Andruff et al., 2009), a one-class model was performed first, and the number of estimated latent classes was increased sequentially until additional classes no longer improved the models fit. When the best model was identified, each class was named based on its visual pattern of trajectory. A posterior probability was computed for each participant to evaluate their membership to each of the latent classes. The lcmm assigned participants exclusively to that class for which the highest probability was obtained.

A successful classification of the participants was accepted above 0.7 and preferentially the closer the posterior probability was to 1. The best-fit model was selected according to the following criteria: (1) If the Bayesian information criterion (BIC; Schwarz, 1978), Akaike information criterion (AIC; Akaike, 1998) and negative log-likelihood were low; (2) If the mean of the posterior probabilities of the individuals classified in each latent class was above 0.7; and (3) There were no less than 10% of the total number of participants allotted to a single trajectory class.

All models were run for a maximum of 15 iterations from 30 vectors of initial values to avoid convergence to local maxima.

The benefit of the lcmm is that it links multiple measurements (TMD, PA, NA, and LLMS) within one model, allowing the participants to be classified into latent classes (subpopulations) based on multiple indicators.

## Results

The results of the lcmm classifications of the participants responses are displayed above in Tables 3, 4.

**TABLE 3:** Latent class mixed model (lcmm) results of the model fitting process for TMD, NA, and LLMS.

No. of latent classes	Polynomial degree	Log-Lik	AIC	BIC	Entropy	% Participants per class	Mean posterior probabilities
1	Linear	−6518	13065	13092	–	100	na
1	Quadratic	−6518	13067	13096	–	100	na
1	Cubic	−6511	13057	13087	–	100	na
2	Linear	−6482	13003	13039	0.801	79.5/20.5	0.93/0.97
2	Quadratic	−6475	12994	13034	0.817	63.6/36.4	0.95/0.95
2	Cubic	−6468	12983	13026	0.814	61.4/38.6	0.96/0.92
3	Linear	−6453	12956	13001	0.757	59.1/15.9/25	0.89/0.94/0.87
3	Quadratic	−6449	12954	13004	0.867	20.5/70.5/9.1	0.98/0.93/0.99
3	Cubic	−6436	12933	12989	0.871	27.3/63.6/9.1	0.9/0.95/0.99
4	Linear	−6435	12929	12983	0.831	9.1/18.2/54.5/18.2	0.99/0.91/0.88/0.96
4	Quadratic	−6412	12892	12953	0.878	29.5/9.1/54.5/6.8	0.86/0.98/0.95/0.99
**4**	**Cubic**	−**6402**	**12881**	**12949**	**0.871**	**13.6/54.5/20.5/11.4**	**1/0.92/0.92/0.94**
5	Linear	−6402	12874	12936	0.887	6.8/15.9/59.1/6.8/11.4	1/0.96/0.92/0.99/0.88
5	Quadratic	−6397	12875	12946	0.880	4.5/25/54.5/9.1/6.8	1/0.88/0.91/0.98/1
5	Cubic	–	–	–	–	Non-convergence	Non-convergence

*Data presented are: the number of latent classes considered, the polynomial form of the model, the maximum Log-Likelihood (Log-Lik), Akaike information criterion (AIC), the Bayesian Information Criterion (BIC), entropy, the posterior classification of participants into each class (%), the mean of posterior probabilities in each latent class. The model chosen to categorise the data and make inferences from in the current manuscript is highlighted in red.*

**TABLE 4:** Latent class mixed models (lcmm) results of model fitting process for PA.

No. of latent classes	Polynomial degree	Log-Lik	AIC	BIC	Entropy	% Participants per class	Mean posterior probabilities
1	Linear	−1795	3602	3613	–	100	na
1	Quadratic	−1782	3577	3590	–	100	na
1	Cubic	−1782	3579	3593	–	100	na
2	Linear	−1782	3586	3606	0.729	84.1/15.9	0.92/0.92
**2**	**Quadratic**	−**1766**	**3558**	**3581**	**0.761**	**15.9/84.1**	**0.96/0.93**
2	Cubic	−1762	3555	3582	0.859	86.4/13.6	0.97/0.90
3	Linear	−1771	3574	3603	0.734	36.4/15.9/47.7	0.87/0.84/0.89
3	Quadratic	−1747	3532	3566	0.907	84.1/13.6/2.3	0.97/0.89/1
3	Cubic	−1746	3536	3575	0.898	4.5/13.6/81.8	0.96/0.90/0.97

*Data presented are: the number of latent classes considered, the polynomial form of the model, the maximum Log-Likelihood (Log-Lik), Akaike information criterion (AIC), the Bayesian Information Criterion (BIC), the entropy, the posterior classification of subjects in each class (%), the mean of posterior probabilities in each latent class. The model chosen to categorise the data and make inferences from in the current manuscript is highlighted in red characters.*

Table 3 indicates the process of searching for the best model with lcmm based on TMD, NA, and LLMS (i.e., moving from identifying only 1 class to 2, to 3, etc.). Following the model selection criteria outlined in the section “Statistical Analysis,” a cubic model with four classes was accepted because it had a low BIC and no less than 10% of the total number of participants allotted to any single trajectory class. The detailed parameter estimates for each class in the optimal model are given in Table 5.

**TABLE 5:** The fixed effects in the longitudinal model for TMD, NA, and LLMS.

	Class 1				Class 2				Class 3				Class 4			
	Coefficient	SE	Wald	*p*-value	Coefficient	SE	Wald	*p*-value	Coefficient	SE	Wald	*p*-value	Coefficient	SE	Wald	*p*-value
Intercept	0 *(not estimated)				–0.408	0.580	–0.703	0.482	–0.401	0.661	–0.606	0.545	–0.831	0.780	–1.065	0.287
Day	0.821	0.250	3.288	**0.001**	0.075	0.107	0.698	0.485	0.182	0.186	0.978	0.328	0.589	0.248	2.375	**0.018**
Day^2^	–2.611	0.655	–3.988	**0.000**	–0.478	0.264	–1.811	0.070	–0.169	0.450	–0.375	0.708	–0.511	0.570	–0.896	0.370
Day^3^	0.176	0.044	4.049	**0.000**	0.039	0.018	2.208	**0.027**	0.000	0.030	0.014	0.989	0.011	0.038	0.300	0.764
F_*I*_O_2_	1.707	0.344	4.970	**0.000**	0.160	0.105	1.521	0.128	1.253	0.253	4.964	**0.000**	1.621	0.317	5.113	**0.000**
Activity	0.393	0.232	1.696	0.090	0.116	0.100	1.153	0.249	–1.397	0.274	–5.093	**0.000**	0.075	0.243	0.311	0.756

*Not estimated, the mean intercept in the first class is constrained to 0. Statistical significance is indicated in bold.

**FIGURE 1:**
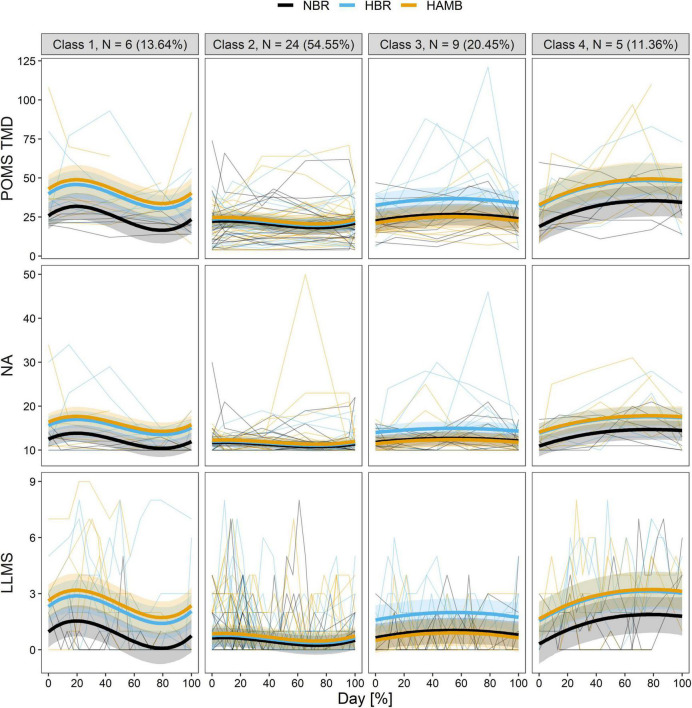
The predicted trajectories of the four distinct classes in the longitudinal total mood disturbance of profile of mood states (POMS TMD; top row), negative affect (NA; middle row), and Lake Louis mountain sickness (LLMS; bottom row) during experiment of each of the interventions (HAMB, hypoxic ambulatory confinement; HBR, hypoxic bed rest; and NBR, normobaric normoxic bed rest). Bold lines show the class-specific mean predicted levels as a function of the percentage of duration, and the ribbons represent the corresponding 95% CI. Thin lines depict individual scores.

Figure 1 depicts the predicted average trajectories of TMD, PN, and LLMS in each class, as well as the actual measured values of participants classified into those classes.

Class 1 “descending” (13.64%; *n* = 6) was characterised by an increase in the first half of the intervention period, followed by a decrease then by a slight increase during the recovery period. Hypoxia was significant as a covariate (*p* < 0.001), with HBR and HAMB scoring higher than NBR. Activity level did not have a significant effect on this class (*p* = 0.090).

Class 2 “flat” (54.55%; *n* = 24) maintained a flat trajectory with low values and little variation in the scores from pre-intervention values. The majority of participants are included in this class. The effects of hypoxia and activity level were both insignificant (F_*I*_O_2_: *p* = 0.128; Activity: *p* = 0.249), as such there were no differences in scores between intervention conditions.

Class 3 is “somewhat flat” like class 2 (20.45%, *n* = 9) and was classified by no significant time variables (Day: *p* = 0.328; Day^2^: *p* = 0.708; Day^3^: *p* = 0.989). The effects of hypoxia and activity level were significant (F_*I*_O_2_: *p* < 0.001; Activity: *p* < 0.001), and according to the coefficients of each variable, hypoxia had the effect of increasing the score while activity level had the effect of decreasing the score. HBR indicated higher scores than the NBR and HAMB conditions.

Class 4 “ascending” (11.36%; *n* = 5) displayed a trajectory in which scores increased substantially early in the intervention and remained high. Hypoxia was significant as a covariate (*p* < 0.001), with HBR and HAMB scoring higher than NBR. The activity level was not significant as a variable (*p* = 0.756).

**FIGURE 2:**
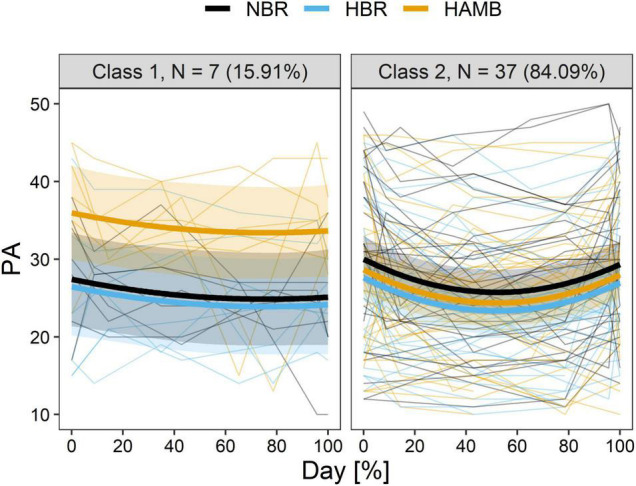
The predicted trajectory of two distinct classes in longitudinal positive affect (PA) during experiment of each of the interventions (HAMB, hypoxic ambulatory confinement; HBR, hypoxic bed rest; and NBR, normobaric normoxic bed rest). Bold lines show class-specific mean predicted levels as a function of percentage of duration, and the ribbons represent the corresponding 95% CI. Thin lines depict individual scores.

The search process of the optimal latent class model for PA is presented in Table 4. During the model selection process, the results of AIC and BIC were in conflict, thereafter BIC was given priority (Nylund et al., 2007; van de Schoot et al., 2017). According to the model selection criteria, a quadratic model with two classes was accepted. Table 6 lists the parameter estimates for each class according to the best model. The fact that the participants PA responses could be categorised into two trajectories rather than four (TMD, NA, and LLMS) suggests that there is less individual variation present in positive emotions trajectories. However, there is still considerable variation in the actual response (please see Figure 2).

**TABLE 6:** The fixed effects in the longitudinal model for PA.

	Class 1				Class 2			
	Coefficient	SE	Wald	*p*-value	Coefficient	SE	Wald	*p*-value
Intercept	27.420	3.089	8.876	**0.000**	30.006	1.509	19.882	**0.000**
Day	−0.675	0.575	−1.175	0.240	−1.640	0.295	−5.557	**0.000**
Day^2^	0.444	0.539	0.824	0.410	1.573	0.283	5.556	**0.000**
F_*I*_O_2_	−0.968	1.479	−0.655	0.513	−2.391	0.789	−3.032	**0.002**
Activity	9.515	1.629	5.842	**0.000**	1.029	0.738	1.395	0.163

*Statistical significance is indicated in bold.*

Figure 2 depicts the predicted mean trajectory of the PA and the measured values for the individual participants allotted to each class.

Class 1 “flat” (15.91%; *n* = 7) exhibited a flat slightly descending trajectory with no significant effect of time or hypoxia (Day: *p* = 0.240; Day^2^: *p* = 0.410; F_*I*_O_2_: *p* = 0.513). However, there was a significant effect of activity level (*p* < 0.001) and higher PA scores were reported in HAMB than in either HBR or NBR.

Class 2 “U-shape” (84.09%; *n* = 37) categorised more than 80% of the total number of participants. The trajectory of PA reported, decreased in the first half of the intervention period and tended to increase in the second half and into the recovery period. While hypoxia significantly decreased PA (*p* = 0.002), activity level did not significantly influence the PA of these participants (*p* = 0.163).

## Discussion

The principle finding of the current study is that significant individual variation exists in the emotional or psychological response to hypoxia and bed rest. When the data is analysed as means, this variation or noise may distract from drawing concrete inferences on the data. Additionally, these findings provide an insight to the inconsistent emotional changes reported in previous bed rest studies. The present study analysed the combined data from three bed rest projects and classified the participants based on the trajectories of their psychological mood state into four distinct classes for TMD, NA, and LLMS, and into two classes for PA. The largest number of participants were classified as having a flat trajectory in both negative and positive models. It is noteworthy that more than half of the participants were able to maintain a flat mental state, as negative emotions increase under stressful conditions. A significant effect of hypoxia on negative affect was found in about half of the participants (Class 1, 3, and 4), importantly, for the remaining half (class 2 “flat”), hypoxia was not significant. There are large individual differences in physical adaptation to the hypoxic environment (Chapman et al., 1998; Townsend et al., 2002), and the current study indicates that psychological responses also vary among individuals.

### General Effects

Emotional tension resulting from an unfamiliar, adverse or demanding situation may result in stress. The results of such a situation may manifest in a heightened state of arousal (positive outcome) or more negatively, with an increase of anxiety, fear, or hostility (negative outcome). Typical stressors are external, usually environmental (temperature, hypoxia, bed rest, space flight) or internal and psychological (relationships or social encounters). Stress is a normal reaction ubiquitously experienced by humans; however, the resultant emotional profile may differ. During endeavours, such as space flight and mountaineering, particularly with increased elevation, there is undoubtedly a heightened level of stress related to the potential success of the mission and the ever-present threat of an accident. Further, the potential effect of increased levels of hypoxia on the individual and group dynamics is currently unknown. There is an importance to examining the interplay between mood dimensions in hypoxic environments and demonstrated significant interaction between psychomotor ability, mental efficiency and the profile of mood state (POMS) mood dimensions, including confusion, fatigue and tension (Bolmont et al., 2000) along with how each individual is behaving or adapting to the current stressor. While we previously reported (Stavrou et al., 2015) that activity (HAMB) would counteract the negative aspect of hypoxia, the current results indicate that this was only the case in nine participants or 20% of those enrolled in the programme. The effect of activity on decreasing negative emotions was found only in class 3, with no significant effect in the other 80% of participants. The positive affect model showed similar results, with only 15% of participants indicating an increase in positive affect from the activity (please see class 1, Figure 2). Thus, the types of physical activity on offer to the participants may not have been desirable, or the required commitment to a physical activity session was not tolerated well by the majority of the participants. Appropriate exercise prescription beyond a general mimicry of physical activity may provide more emotional relief in hypoxia. However, given the current results, this should be specific and individually tailored.

In most bed rest studies, participants’ emotions changed negatively (Ishizaki et al., 2002; Liu et al., 2012, 2015; Stavrou et al., 2015, 2018a), although there are reports that participants’ emotions did not change in a 15-day restraint experiment (Zhao et al., 2011). Our results indicate that the 10- and 21-day durations were not significant as covariates predicting differences in psychological state, and that individual variability was a substantial factor in the inconsistency between previous studies. Thus, duration was not a factor in the response which was driven by the intervention and intrinsic factors. Differences in emotional changes between participants may have been due to different emotional regulation strategies. Emotional regulation is the ability to respond throughout the duration of any experience to its ongoing demands (Cole et al., 1994). To worry by focussing on only the potentially negative events in the future, serves to aid in the upregulation of negative emotion (Campbell-Sills and Barlow, 2007). Before taking part in the current experiment, participants of the “flat” class were unlikely to have been anxious about what life would be like during the period of confinement, they coped well with the intervention and were not negatively affected by hypoxia or inactivity. The “ascending” class experienced more stress than they had imagined or could cope with and so their negative emotions increased throughout the intervention.

It has been reported that confinement experiments often result in a lack of emotional upheaval in the participants. At the Concordia Station in Antarctica, the over wintering participants may enter a state of psychological hibernation as a stress coping mechanism (Sandal et al., 2018). Participants are released from undesirable thoughts and situations by diverting their attention from certain thoughts and mental images to other contents (Campbell-Sills and Barlow, 2007). Although, thought suppression and psychological hibernation can only occur after a long period of confinement, up to several months, this does not apply to the present experiment, which involved up to a few weeks of confinement.

It is well established that as we age, we become more skilful at regulating our emotions, or not allowing innocuous events to overload us emotionally, we may conserve energy for the tasks that really do challenge us. Labouvie-Vief et al. (2010) conclude that middle aged adults experience more positive affect and less negative affect than younger adults. When older adults are exposed to unpleasant stimuli, they are able to regulate their emotional responses in such a way as to avoid negative confrontations (Charles and Carstensen, 2008). Since participants in both 10 days and 21 days confinement were young adults in their twenties, there appears to be no age-related benefits in emotion regulation strategies. However, astronauts are typically 45 years of age and older when they fly, therefore it’s likely that these age-related emotional regulation patterns come into play for that demographic, combined with extensive specialty training. It is possible that astronauts would therefore present with a different set of trajectories, albeit still displaying individual variation in their responses.

### The Effect of Sex

Existing studies have reported that females have higher anxiety (Feingold, 1994; Kessler et al., 1994; McLean et al., 2011), depression (Hathaway and McKinley, 1989; Cyranowski et al., 2000; Whissell, 2003; Ford and Erlinger, 2004; Albert, 2015), and fatigue than their male counterparts (Verbrugge, 1985; van Wijk and Kolk, 1997; Miaskowski, 2004). On the other hand, outside of a bed rest study, it is typical for males to score higher than a parallel group of females on both state anger and trait anger (Spielberger, 1999; Whissell, 2003). However, with regard to the present results, the sex variable was not significant as a covariate of lcmm, and there were no sex differences in psychological state. There are potentially several reasons to believe that changes in sleep as a result of bed rest and hypoxia can invalidate sex differences in emotion. Indeed, Rojc et al. (2014) found that in the LunHab study, exposure to both hypoxia and bed rest resulted in greater sleep fragmentation due to more awakenings throughout the night. A separate study at the German Antarctic Station, Neumayer, indicates that confinement experiments may lead to poor sleep quality in women (Steinach et al., 2016). Sleep deprivation is associated with an increased emotional response to negative and stressful stimuli (Walker, 2009). As a result of the deterioration in sleep quality in both women and men, negative emotions may have increased and sex differences in the emotional state may have disappeared.

### Source of Variation

There may be several sources of variation seen within the results. This could be the precision and accuracy of the measurement tool, the POMS and PANAS instruments themselves or the Planica environment and how all the staff and participants interact. Due to the nature of the protocol, the participants in the present study were subject to confinement and limited to social interaction within the participant pool. Both of these factors may contribute to the enhancement of negative mood. Results from normoxic bed rest studies, in which participants are inactive and their lower limbs unloaded to induce the musculoskeletal atrophy observed in astronauts during space missions, have shown that cortisol levels remain stable during 14 days, 17 days, or 20 days of bed rest (Ishizaki et al., 1994; Ferrando et al., 1996; Millet et al., 2001). However, others have found that cortisol increased concomitantly with depression after 20 days of bed rest (Ishizaki et al., 2000, 2002). It is possible that with modifications to the social interaction of participants, both mood and cortisol remain unchanged after 20 days of bed rest (Ishizaki et al., 2004). Thus, socialisation is an important factor along with group dynamics; however, whilst it can have beneficial effects, it must also be considered that the social interaction and information provided by others within the social group can result in an increase in the stress response (Ishizaki et al., 2004; Gallagher et al., 2014). It is pertinent to consider that some participants enjoyed and some disliked this setup, along with the potential for differing experiences in different rooms or shared spaces.

Finally, one must consider that the process of physiological adaptation to an environmental stressor also varies among participants. How the variance of those physiological adaptations or mal adaptations combine to affect the psychological response is currently unknown.

## Conclusion

The use of the latent classes mixed modelling analysis provides clear evidence that presenting the psychological response to bed rest and hypoxia as means and standard deviations is not appropriate. The lcmm approach categorised the participants into four distinct classes based on the trajectory of their responses throughout the interventions. Individual participants exhibit a range of emotional responses to bed rest and hypoxia, which may be influenced by sex, activity level and intervention duration, along with inherent factors as yet unidentified. Lcmm provides an opportunity for clear interpretation of the data by not presenting results as means and disregarding the responses of 50% of the participant group.

## Data Availability Statement

The original contributions presented in the study are included in the article/supplementary material, further inquiries can be directed to the corresponding author.

## Ethics Statement

The studies involving human participants were reviewed and approved by the National Committee for Medical Ethics at the Ministry of Health of the Republic of Slovenia. The patients/participants provided their written informed consent to participate in this study.

## Author Contributions

ACM and IM contributed to the conception and design of the study as well as collected the data. KT performed the statistical analysis and prepared a first draft of the manuscript. KT, IM, and ACM wrote individual sections culminating in the final draft. All authors contributed to the manuscripts critical revision, have read and approved the submitted version.

## Conflict of Interest

The authors declare that the research was conducted in the absence of any commercial or financial relationships that could be construed as a potential conflict of interest.

## Publisher’s Note

All claims expressed in this article are solely those of the authors and do not necessarily represent those of their affiliated organizations, or those of the publisher, the editors and the reviewers. Any product that may be evaluated in this article, or claim that may be made by its manufacturer, is not guaranteed or endorsed by the publisher.
